# Perspectives of infant active play: a qualitative comparison of working versus stay-at-home parents

**DOI:** 10.1186/s12889-021-10286-x

**Published:** 2021-01-30

**Authors:** Kailey Snyder, John P. Rech, Kim Masuda, Danae Dinkel

**Affiliations:** 1 School of Pharmacy and Health Professions, Creighton University, 2500 California Plaza, Omaha, NE 68178 USA; 2 School of Health & Kinesiology, University of Nebraska at Omaha, 6001 Dodge Street, Omaha, NE 68182 USA

**Keywords:** Infant, Parent, Active play, Physical activity, Working mother, Stay-at-home mother

## Abstract

**Background:**

Parents play a key role in infant’s development through their interactions and the type of environment they provide to promote active play. The amount of time parents are able to spend with their infant is dependent on their working status, yet few studies have explored parent perception of their infant’s active play by working status. The purpose of this study was to explore parent perception of active play and compare responses between working and stay-at-home parents.

**Methods:**

Twenty-nine parents participated in this qualitative study by completing a one-time, in-person semi-structured interview based on the Theory of Planned Behavior. Themes were developed and compared based on parental working status using a directed content analysis approach.

**Results:**

All parents believed active play could have a positive effect on their child’s development through physical, social and emotional, cognitive, and/or language and communication development. However, stay-at-home parents reported a broader impact of active play across these domains; whereas working parents most often referenced active play as impacting infant’s physical development. Social and emotional interactions were the highest reported form of active play among all parents. Additionally, all parents described similar barriers to increasing the time for active play. The most commonly reported barrier for all parents was time or schedule followed by care needs of the infant, environmental concerns, and need for restrictive devices (e.g., car seats). More stay-at-home parents than working parents reported the care needs of the infant as being a barrier. Recommendations for active play were not widely known amongst all parents, with a higher percentage of working parents reporting they would desire advice from a healthcare provider.

**Conclusions:**

Working status of parents appears to have implications on perceptions of active play which in turn may influence infants’ development. Future studies should objectively assess the impact of parents’ working status on infant development and explore how gender of the parent may serve as a confounding variable.

**Supplementary Information:**

The online version contains supplementary material available at 10.1186/s12889-021-10286-x.

## Background

The first year of life is critical to a child’s development [1]. Parents undoubtedly play a key role in infants’ development through their interactions and the type of environment they provide for their child [2, 3]. However, the amount of time parents are able to spend with their infant during this development period is greatly influenced by their status of being a working or stay-at-home parent.

Globally, several countries are seeing a rise in the percentage of working mothers [4–6].

With more parents working, research has begun to examine how employment may impact various domains of development. For example, infant cognitive development has been positively associated with a mother working; however, this is suggested to be tied to increased resources available due to being in a higher income household [7]. Related to physical development, some studies have found a negative impact on children’s motor skills when mothers returned to work within the first year of life [8, 9]. Conversely, research suggests infant social, emotional, communication skills or physical development do not differ based on parental work status [10–12]. Given the variation in findings across different developmental domains more research is needed to elucidate how parent-infant interaction could influence an infant’s development [13–16].

One pathway for parent interaction and influencing a child’s development is through active play [17, 18]. Active play for infants can be defined as opportunities to be active several times a day in a variety of ways such as through interactive floor-based play [17]. Importantly, active play can be a positive influence on a child’s cognitive, physical, social, emotional, and language and communication development [12–19].

Governmental organizations all over the world have begun to recognize the importance of active play and have provided recommendations for parents and caregivers. In the United States, the American Academy of Pediatrics recommends that there is dedicated time every day for active play while limiting the amount of time spent in items that restrict movement such as car seats, strollers, and bouncy seats and that infants engage in 30–60 min of tummy time per day [19, 20]. Other countries such as Australia’s Department of Health have provided additional guidance including 1) being physically active several times a day in a variety of ways including supervised interactive floor-based play, 2) 30 min of tummy time for those not yet mobile; and 3) limit time in items that restrain movement for no more than 1 h at a time [21]. Despite these guidelines, few studies are examining infant active play and previous research has primarily focused on how often infants are placed in “tummy” time. Importantly, in the United States only half of infants are achieving the American Academy of Pediatrics tummy time recommendations of 30–60 min per day [20].

Given the limited research conducted on active play, especially with a wider focus beyond tummy time, and the influence parental employment has on infant development more research is needed in these areas. Further most studies have focused primarily on maternal employment influences and more recent studies including fathers are needed [22]. Therefore, the purpose of this study was to explore parents’ perceptions of active play and compare responses between working and stay-at-home parents.

## Methods

Parents were recruited to participate in a larger study assessing parent and infant play interactions. Additional details regarding the full methodology of this study can be found elsewhere [3]. Specific to this qualitative content analysis, semi-structured in-person interviews were conducted with all participants in the Fall of 2018 thru Spring of 2019. This study was approved by a University Affiliated Institutional Review Board.

### Participant recruitment and selection

Purposive sampling was utilized to recruit study participants as part of the larger study [23]. Recruitment took place via flyers at maternal/child friendly businesses, sharing study information on maternal support *Facebook* groups, and word of mouth. If interested in participating, parents were directed to an eligibility survey online through Qualtrics survey software [24]. If eligible, based on parental age (> 19 years), age of infant (≥6 months), and ability of infant to sit independently (defined as being able to sit without assistance for at least 10 s), meeting times to collect data were scheduled in the comfort of the participant’s home. A total of 38 individuals completed the eligibility survey. Of these 38, 32 met eligibility criteria, however, 3 were unavailable when research personnel followed up to schedule an appointment. A total of 29 parent/infant dyads were included in the study.

### Instruments

A semi-structured interview guide was developed by two trained female qualitative researchers employed in academia (MS & PhD). A total of 31 semi-structured questions, consisting of both open and closed questions were developed based on constructs of the Theory of Planned Behavior (TPB). The TPB provides a model to better understand the connection between beliefs and behavior [25]. Specifically the guide focused on the constructs of *attitude* (a person’s favorable or unfavorable perceptions of a behavior), *perceived behavioral control* (a person’s belief in their ability to take part in the behaviors), and *subjective norms* (a person’s belief on other peoples’ thoughts on the behavior). Table 1 provides example questions of each construct. The interview guide was pilot tested with two parents and small wording changes were conducted after the pilot interviews were completed to enhance clarity.

**Table 1:** Example interview questions asked to parents based on theory of planned behavior model

Construct	Questions
Attitude	1. Tell me about the feeling or thoughts you associate with when you hear the term “active play.” 2. Tell me about your feelings or thoughts about your child’s current weight.
Perceived Behavioral Control	3. What recommendations for active play and sedentary time for infants have you heard previously? a. Who did you hear this from? b. Do you follow these guidelines? Why or why not? 4. What control do you think you have over your child’s weight?
Subjective Norms	5. Who would be the person you would most listen to when it comes to your child’s active play? 6. Who or what would you turn for resources or advice regarding your child’s weight?

### Data collection

Each data collection session consisted of two researchers visiting the parent/infant dyad in their home. Prior to participation the parent reviewed and signed a consent form which discussed the risks of participation. Parents were also asked if they had any questions prior to the data collection taking place and reminded that they could halt study participation at any time.

Upon parents’ written consent, parents completed a demographic survey that provided the following information about themselves: age, weight, height, household income, education and current employment. As a part of the larger study, infants and parents completed a variety of motor development and play measures described elsewhere [3]. After the survey and other assessments were completed the interview was conducted. All interviews were conducted by KS and audio-recorded and lasted approximately 20 min. Each parent completed one interview and no repeat interviews were carried out.

### Data analysis

Interviews were transcribed verbatim into a Word document and uploaded using NVivo 12, a qualitative software [26]. For the purpose of this analysis parents were compared based on how they self-identified in a demographic survey, either as “working” if they were not with the child during typical care hours of 8–5 pm or “stay-at-home” if they were the primary caretaker during these hours [27].

A directed content analysis approach was used to develop the coding scheme [28]. Therefore, the coding scheme was developed by two professional students (JR, KM) being trained in qualitative methodology under the direction of two PhD trained qualitative researchers (DD, KS). First, the two students individually read all the interview transcripts multiple times to identify trends and themes in order to deductively develop parent codes according to the TPB. Next, an inductive strategy was used to create child codes underneath the respective parent codes in order to uncover themes in participants responses. The codes that were developed for the perceived impact of and engagement in active play aligned with the Center for Disease Control and Prevention’s (CDC) developmental milestones for infants 6–9 months, which include general areas of cognitive, physical, social and emotional, and language and communication development [29]. A codebook was developed to provide an outline of the coding scheme and definition of codes.

Throughout the coding process a combination of peer debriefing and thick description was used to ensure data validation [30–33]. The two students separately reviewed and coded 9 of the 29 interviews, selected at random. The coding of the first 9 interviews were then compared for discrepancies by all authors. Consensus was reached through open discussion between all authors. The two students then coded an additional 10 interviews and a similar process was used to come to a consensus on all coding. Finally, the remaining 10 interviews were then coded. A total of four meetings between all authors took place before coding was considered complete. After coding was complete, differences were compared between working and stay-at-home parents based on percentage responses to each code. All four authors met a final time to review the similarities and differences between the two groups.

## Results

The demographics of the participants can be found in Table 2. Of the 29 parents, 16 were considered to be a stay-at-home parent, whereas 13 were considered to be a working parent. The majority of the parents who participated in the interviews were females (89.7%).

**Table 2:** Parent Socio-demographic Information

Characteristics	Stay-at-home *n* = 16 (n/%)	Working *n* = 13 (n/%)
Parent’s Gender		
Male	0 (0.0)	3 (45.5)
Female	16 (100.0)	10 (54.5)
Infant’s Gender		
Male	6 (37.5)	8 (61.5)
Female	10 (62.5)	5 (38.5)
Parent’s Age		
Age (years)	31.63 ± 4.87	30.46 ± 4.75
Parent’s Education (n, %)		
High school degree or less	1 (6.3)	2 (15.4)
Some college	4 (25.0)	1 (7.7)
Bachelor’s degree	6 (37.5)	4 (30.8)
Master’s degree	4 (25.0)	3 (23.1)
Doctorate	1 (6.3)	3 (23.1)
Annual Income, USD (n, %)		
Less than $60,000 per year	4 (25.0)	1 (7.7)
$61,000–$100,000	4 (25.0)	4 (30.8)
$101,000 per year and above	8 (50.0)	8 (61.5)
Parent’s Ethnicity (n, %)		
African American	0 (0.0)	1 (0.0)
White	16 (100.0)	12 (100.0)
Parent’s BMI (mean, sd)		
BMI	26.70 ± 4.98	27.06 ± 7.38

Table 3 provides an overview of the themes broken down by the proportion of working and stay-at-home parents who responded within each theme.

**Table 3:** Overview of Results

Theme	All parents	Stay-at-home parents	Working parents	Sample quote
**Perception of active play**				
Interaction with others	62.07%	68.75%	53.85%	“I think active play is when you are involved in your kid when you are actually playing with them. Interacting with them back and forth.”
Individual play	37.93%	31.25%	46.15%	“I would say her being able to like engage with the toys and not just be sitting next to them and not acknowledging them.”
**Perceived impact of active play**				
Physical	72.41%	75%	69.23%	“That’s how she builds her muscles and that’s how she uses them.”
Social and Emotional	65.52%	75%	53.85%	“...I think it gives them better social skills.”
Cognitive	62.07%	75%	46.15%	“I think it promotes curiosity, independence, obviously brain development, it gets them kind of working on the why and how of things.”
Language/Comm.	31.03%	37.5%	23.08%	“...talking to your kid helps with the language development and then eventually the reading and the word recognition, and just the vocabulary.”
**Engagement in active play**				
Physical	68.97%	75%	61.54%	“With her, we try to practice her crawling and walking. Tummy time and rolling over.”
Social and Emotional	86.21%	87.5%	84.62%	“…encouraging his facial recognition. Like smiling, sad, the various.”
Cognitive	62.07%	62.5%	61.54%	“...showing her things, and encouraging her to use things, like hitting the drum, or like showing her what toys are supposed to do. Like you make a ball bounce and you make a car go.”
Language/Comm.	44.83%	50%	38.46%	“...engaging, talking to her, making eye contact. Sometimes she is great about eye contact and then there is other times she is not but I think getting actively going back and forth.”
**Barriers to active play**				
Time or schedule	44.44%	46.67%	41.67%	“The only barrier is just kind of time. Because he has his own schedule.”
Infant care needs	29.63%	40%	16.67%	“…wants to eat you know, is ready for a nap and what not. He is an early bed person.”
Environment	29.63%	33.33%	25%	“...environment, there’s other kids, there’s animals, it’s dirty…”
Restrictive devices	11.11%	6.67%	16.67%	“...it’s easier just to put her in the carrier and put her on my back and um and just kind of keep her hang out with me while they’re doing their play.”
Childcare	3.7%	0%	8.33%	“Daycare is a barrier. Um only he is in-home and even with a center the expectations they kind of try to keep them safe rather than on track at this age.”
None	18.52%	20%	16.67%	“Not at this time.”
**Recommendations for active play**				
Heard of	55.17%	50%	61.54%	Questions were closed ended therefore participant responses were coded as yes or no.
Have not heard of	44.83%	50%	38.46%	
Doctor mentioned	31.03%	25%	61.54%	
Doctor did not mention	68.97%	75%	38.46%	

### Perceptions of active play

When parents were asked their thoughts and feelings about active play, they described active play as either interactions with others (62.1%) or individual play (37.9%). If a parent mentioned interaction with others, they often described active play using words such as showing, engaging, bonding, or learning. Parents who described active play as individual play often defined active play as their infant interacting with toys and/or their environment without explicitly stating if the infant was interacting or playing with someone. Of the stay-at-home parents, 68.8% viewed active play as the infant’s interaction with others whereas 31.3% viewed active play as individual play. For instance, a stay-at-home parent described active play as “…interacting with your child while they are playing, so not having them play independently as much so like rolling the ball back and forth or showing them how things open and shut.” Among working parents, thoughts and feelings of active play was more evenly split between those who stated interaction with others (53.8%) and those who stated active play as individual play (46.2%). For example, a working parent stated active play as “…any time there’s kind of like a purpose, you can tell he is going after a toy or playing with a toy for an increased or set amount of time.”

### Perceived impact of active play

Overall parents viewed active play as primarily having an impact on physical (72.4%); social and emotional (65.5%); and cognitive (62.1%) development. Few parents, regardless of working status, referenced an impact on language and communication development (31.0% of all parents; 37.5% of stay-at-home; 23.1% of working). However, differences in responses between stay-at-home and working parents were found between physical, social and emotional, and cognitive development. Stay-at-home parents reported a broader impact of active play with a majority describing an impact on their child’s physical (75.0%), social and emotional (75.0%), and cognitive (75.0%) development. For example, a stay-at-home parent explained: “Just because when they are really young learning different things, how to grasp, how to hold, how to talk, interact, and you know recognizing voices, you can’t just sit there you know talking straight to them but you are moving stuff around to which helps them follow sounds, move their heads.” Working parents mostly viewed active play as impacting infant’s physical (69.2%) development, more than social and emotional (53.8%) or cognitive (46.2%) development. One working parent stated: “Well I feel like if we are encouraging him to you know stand up by helping him a little bit or uh you know crawl by putting a foot out in front of him that’s kind of motivating him to get moving and we’ll help his development faster than you know if we weren’t doing that.”

### Engagement in active play

When asked about the form of play parents engaged in with their infants, their responses were similar regardless of working status. Overall, 86.2% of parents reported mostly engaging in social or emotional play. One working parent referenced social and emotional play by stating: “Just being more interactive. As opposed to just sitting him down and walking away. Just actually sitting down interacting with him and giving him full attention.” Additionally, 69.0% of parents mentioned physical play, 62.1% mentioned cognitive play, and 44.8% mentioned play that involved language and communication. A stay-at-home parent mentioned all forms of play in her response by stating: “Yeah I mean so we do the standing with arms held, um facial expressions, um just like showing him how things work. What they are, like I said physically moving around with him. Or dancing, singing, doing all that too.”

### Barriers to active play

All parents described similar barriers to increasing the time for active play. The most common barrier for all parents was time or schedule (44.4%), followed by care needs of the infant (29.6%), environmental concerns (29.6%), and restrictive devices (11.1%). A stay-at-home parent made a reference to time or schedule by stating: “I think having another child, makes it more difficult. It depends on what we have going on that day, you know how much time we are going to have at home.” Parents who reported care needs mentioned things such as feeding, changing diapers, or the infant’s mood. Environmental concerns included factors such as safety, cleanliness, “baby proofing” their surroundings, or weather. For example, one working parent said: “I would like to increase probably some more like outside play but my barriers there would be weather and just also some of the like safety around our house.”

The most notable difference between stay-at-home parents and working parents was that stay-at-home parents (40.0%) were more likely to report the care needs of the infant as being a barrier more than working parents (16.7%). For example, a stay-at-home parent mentioned care needs as a barrier by stating: “…it depends on her mood sometimes, sometimes infants don’t have it. There are going to be days when they are not going to be up for much.” Additionally, among all parents, 18.5% reported having no barriers to increasing active play time with hardly any difference in the rate when comparing stay-at-home and working parents.

### Parental influence on active play

Parents were asked who they believe has an influence on their infant’s active play. All stay-at-home parents reported having personal influence on their child’s activity, whereas a slightly lower percentage of working parents (83.3%) mentioned themselves as having influence on their child’s activity. Among the working parents, 33.3% of them reported the childcare provider as having an influence on their infants’ active play. Additional reported influencers of infant active play included other family members (25.9%), such as siblings or grandparents.

### Recommendations for active play

Parents were asked if they had heard of recommendations for active play and/or sedentary time. Half of parents regardless of working status reported that they had not specifically heard of recommendations for active play (55.2%). Of those parents who stated they had heard of active play recommendations (44.8%), almost all of them referred to tummy time. No parents mentioned infant interaction when discussing the recommendations they had heard on active play. When parents were read recommendations on active play and asked about the achievability of these recommendations, 89.7% viewed the American Academy of Pediatrics guidelines as achievable and 92.3% viewed the Australia’s Department of Health guidelines as achievable.

When asked whether their doctor had mentioned active play, a majority of parents (69.0%) reported they had not mentioned or discussed active play. But when the parents were asked who they would listen to about infant active play, a majority of parents (69.0%) reported they would listen to a healthcare provider. However, a higher percentage of working parents (84.6%) stated they would prefer talking about active play with a healthcare provider compared to stay-at-home parents (56.2%). One working parent stated this in regard to her family’s doctor: “I know when they give recommendations it’s not just because they are spouting it out they are always making sure to say this is why we do what we do, this is why we recommend what we recommend.” Stay-at-home parents were also interested in listening to internet or book sources (31.3%), family or friends (25.0%), and guidelines (12.5%). For example, one stay-at-home parent stated: “The internet probably. I think that would be my go to because you get a variety of ideas there.”

## Discussion

The purpose of this study was to explore parents’ perceptions of active play and compare responses between working and stay-at-home parents. Findings demonstrate all parents believed active play had a positive effect on their child’s development in a variety of developmental domains. However, stay-at-home parents were more likely to see active play as impacting physical, social and emotional, and cognitive development compared to working parents who related active play primarily only to physical development. This difference could be due to several factors. First, working parents reported a desire to get information about active play from their healthcare provider. While limited research is available on providers’ knowledge of infant physical activity guidelines, one study in the UK found only 13.6% of providers were able to state the adult physical activity recommendations [34]. Second, parents’ knowledge of guidelines was primarily only in regard to tummy time. This is consistent with previous literature’s focus on tummy time rather than other elements of infant activity such as active play [20]. While tummy time is crucial for avoiding positional plagiocephaly, it is also typically associated with benefits to physical development. Since healthcare providers meet regularly with parents, an important first step may be to ensure that healthcare providers themselves are aware of the active play guidelines for infants. However, our study found the majority of parents reported not discussing active play with their doctor and previous research suggests primary care physicians have a limited amount of time to spend with patients to discuss infant development [35]. Thus, development and/or dissemination of resources which emphasize the multiple developmental domains of active play as well as how parents can engage in active play with their infants is needed [34]. While working parents reported they would like this information from healthcare providers and stay-at-home parents desire information from multiple sources such as the Internet, family and friends, and additional avenues should also be explored.

For example, interprofessional support could be considered by involving pediatric occupational therapists and physical therapists in not only the resource development but also dissemination efforts. Often these individuals are consulted in a reactive manner when a development issue has already been identified [36]. Future research should consider avenues that involve healthcare providers from multiple disciplines to receive education on infant active play as well as to design resources to share with caregivers. Importantly, the development of resources with input from a variety of disciplines as well as dissemination through multiple sources could help to improve the critical need to improve parents’ knowledge of active play recommendations [37].

This lack of knowledge was prevalent within our study as half of parents were not aware of any recommendations for active play. However, when presented with the different active play guidelines, the majority of parents felt the American Academy of Pediatrics and Australia’s Department of Health infant activity guidelines were attainable despite being unaware of any guidelines prior to the interview taking place. This is contrary to other research by Carson and colleagues (2014) who examined parents’ perceptions of meeting the Canadian Sedentary Behavior Guidelines for children 0–4 years. Parents in this study were more aware of the guidelines and felt they were easy to understand but did not find them as feasible [38]. When exploring how to enhance discussions in an interprofessional manner, emphasis should be placed not only on ensuring parents are aware of recommendations but also on how they can achieve these guidelines. Healthcare providers could consider focusing on how to help parents overcome barriers to providing active play including improving self-efficacy, scheduling active play time, encouraging variation in play, encouraging siblings and other caregivers (for working parents especially childcare providers) to help with active play, ensuring it is enjoyable for the parent, and understanding of benefits to the child [20].

One additional issue that warrants attention is the role of the COVID-19 pandemic on parent working status. The pandemic has created an unprecedented shift in the number of parents working from home and work flexibility [39]. This has likely resulted in a change in interaction type and frequency between parents and infants; however, research is needed to elucidate these changes. Importantly, previous research suggests changes in work structure such as working from home and increased flexibility may produce positive benefits for the child [40]. For example, working from home has been associated more frequent mother-child interactions and flexible work scheduling has been associated with increased daytime father-child interactions [41]. This previous research took place prior to the pandemic and did not account for the negative impact working from home with the stress of the pandemic has had on parents mental health [41]. Future research should consider how working from home impacts parents’ perceptions of active play and how these perceptions are influenced by parents’ mental well-being.

This study did come with limitations that should be identified. First, our study was limited by having a predominantly white, middle class, female sample. In order to gain a better understanding of both mother and father perceptions of active play, recruitment efforts should focus on attaining father participants to explore potential differences between mothers and fathers. More research is needed exploring the influence of the gender of the parent [42]. Additionally, a more diverse sample by income and race/ethnicity could further shed light on the replicability of these findings. Although interviewing techniques, such as the validation of questions and the use of probing, were initiated, the potential for biases may still exist such as social desirability and recall bias which may hinder the accuracy to which interview questions were answered [43]. Finally, the cross-sectional nature of this study is only capturing parental perceptions at one point in time. Tracking perceptions longitudinally would provide a better understanding of how parents views of active play change as their child ages.

## Conclusion

Overall, our study is one of few studies to explore parents’ perceptions of active play among infants with a wider definition outside of tummy time and the only study, to our knowledge, to compare parent perceptions based on working status. This is an area that is becoming increasingly important given the workplace shift happening related to the COVID-19 pandemic and number of women in the workforce. Findings demonstrate differences in parental perception of active play based on working status. Specifically, working parents appear to desire activity play education from healthcare providers and associate active play primarily with physical development. Conversely, stay-at-home parents see active play as beneficial across several developmental domains and desire resources from a variety of sources. Importantly, few parents are aware of active play recommendations regardless of working status. Future research should explore how perceptions have shifted based on the increase in parents working from home due to the pandemic as well as how to quantify if these perceptions influence infant development.

## Supplementary Information

**Additional file 1.** Interview Guide. The interview guide utilized in this study has been included in its entirety as a supplementary file to this manuscript.

## Data Availability

The datasets used and/or analyzed during the current study are available from the corresponding author on reasonable request.

## References

[1] Winston R, Chicot R (2016). The importance of early bonding on the long-term mental health and resilience of children. London J Prim Care.

[2] Pereira KR, Valentini NC, Saccani R (2016). Brazilian infant motor and cognitive development: longitudinal influence of risk factors. Pediatr Int.

[3] Dinkel D, Snyder K (2020). Exploring gender differences in infant motor development related to parent’s promotion of play. Infant Behav Dev.

[4] Big rise in number of working mothers. BBC News, London. 2017. https://www.bbc.com/news/business-41399493. Accessed 28 Jul 2020.

[5] Work and family. Australian Institute of Family Studies, Melbourne. 2016. https://aifs.gov.au/facts-and-figures/work-and-family. Accessed 10 Nov 2020.

[6] Kazakova Y. Childcare availability and maternal labour supply in Russia: Institute for Social and Economic Research, University; 2019. https://www.iser.essex.ac.uk/research/publications/working-papers/iser/2019-11.pdf.

[7] Kim J, Wickrama KAS (2014). Mothers’ working status and infant development: mediational processes. J Fam Issues.

[8] Sherlock R, Synnes A, Koehoorn M (2008). Working mothers and early childhood outcomes: lessons from the Canadian National Longitudinal study on children and youth. Early Hum Dev.

[9] Wei CF, Chen MH, Lin CC, Guo YL, Lin SJ, Liao HF (2019). Association between maternal shift work and infant neurodevelopmental outcomes: results from the Taiwan birth cohort study with propensity-score-matching analysis. Int J Epidemiol.

[10] Hock E (1980). Working and nonworking mothers and their infants: a comparative study of maternal caregiving characteristics and infant social behavior. Merrill Palmer Quarte Behav Dev.

[11] Brooks-Gunn J, Han WJ, Waldfogel J (2010). First-year maternal employment and child development in the first seven years. Monogr Soc Res Child Dev.

[12] Borba LS, Pereira KRG, Valentini NC. Motor and cognitive development predictors of infants of adolescents and adults mothers. J Phys Educ. 2017;28:2448–55.

[13] Silva SD, Flôres FS, Corrêa SL, Cordovil R, Copetti F (2017). Mother’s perception of children’s motor development in southern Brazil. Percept Mot Skills.

[14] Rocha NACF, Dos Santos Silva FP, Dos Santos MM, Dusing SC (2020). Impact of mother–infant interaction on development during the first year of life: a systematic review. J Child Health Care.

[15] Hesketh KD, Hinkley T, Campbel KJ (2012). Children’s physical activity and screen time: qualitative comparison of views of parents of infants and preschool children. Int J Behav Nutr Phys Act.

[16] Koren A, Kahn-D'angelo L, Reece SM, Gore R (2019). Examining childhood obesity from infancy: the relationship between tummy time, infant BMI-z, weight gain, and motor development—an exploratory study. J Pediatr Health Care.

[17] Guidelines on physical activity, sedentary behaviour and sleep for children under 5 years of age. World Health Organization, Washington. 2019. https://apps.who.int/iris/handle/10665/311664. Accessed 28 Jul 2020. 31091057

[18] Timmons BW, LeBlanc AG, Carson V, Connor Gorber S, Dillman C, Janssen I (2012). Systematic review of physical activity and health in the early years (aged 0–4 years). Appl Physiol Nutr Metab.

[19] Infant Physical Activity. American Academy of Pediatrics, Itasca. 2020. https://www.aap.org/en-us/advocacy-and-policy/aap-health-initiatives/HALF-Implementation-Guide/Age-Specific-Content/Pages/Infant-Physical-Acticity.aspx. Accessed 28 Jul 2020.

[20] Felzer-Kim IT, Erickson K, Adkins C, Hauck JL. Wakeful prone “tummy time” during infancy: how can we help parents? Phys Occup Ther Pediatr. 2020;40(6):651–68. 10.1080/01942638.2020.1742847. 10.1080/01942638.2020.1742847 32192403

[21] Department of Health (2017). Australian 24-hour movement guidelines for the early years (Birth to 5 Years): an integration of physical activity, sedentary behaviour, and sleep.

[22] Berrigan MN, Schoppe-Sullivan SJ, Dush CMK. Moving beyond access: predictors of maternity and paternity leave duration in the United States. Sex Roles. 2020;5:1–14.

[23] Gentles SJ, Charles C, Ploeg J, McKibbon KA (2015). Sampling in qualitative research: insights from an overview of the methods literature. Qual Rep.

[24] Qualtrics Survey Software (2020). Qualtrics XM, Provo.

[25] Montaño DE, Kasprzyk D (2015). Theory of reasoned action, theory of planned behavior, and the integrated behavioral model. Health Behav: Theory, Res Pract.

[26] QSR International Pty Ltd. (2020) NVivo (released in March 2020), https://www.qsrinternational.com/nvivo-qualitative-data-analysis-software/home.

[27] Lindsay S (2019). Five approaches to qualitative comparison groups in health research: a scoping review. Qual Health Res.

[28] Hsieh HF, Shannon SE (2005). Three approaches to qualitative content analysis. Qual Health Res.

[29] National Center on Birth Defects and Developmental Disabilities (2020). Important milestones: your baby by six months.

[30] Geertz C (1973). Thick description: toward an interpretive theory of culture. Turning Points Qual Res: Tying knots Handkerchief.

[31] Henry P (2015). Rigor in qualitative research: promoting quality in social science research. Res J Recent Sci ISSN.

[32] Long T, Johnson M (2000). Rigour. Reliability and validity in qualitative research. Clin Eff Nurs.

[33] Ponterotto JG (2006). Brief note on the origins, evolution, and meaning of the qualitative research concept thick description. Qual Rep.

[34] Cuthill JA, Shaw M (2019). Questionnaire survey assessing the leisure-time physical activity of hospital doctors and awareness of UK physical activity recommendations. BMJ Open Sport Exerc Med.

[35] Dudek-Shriber L, Zelazny S (2007). The effects of prone positioning on the quality and acquisition of developmental milestones in four-month-old infants. Pediatr Phys Ther.

[36] Harrison M, Jones P, Sharif I, Di Guglielmo MD (2017). General pediatrician-staffed behavioral/developmental access clinic decreases time to evaluation of early childhood developmental disorders. J Dev Behav Pediatr.

[37] Willumsen J, Bull F (2020). Development of WHO guidelines on physical activity, sedentary behavior, and sleep for children less than 5 years of age. J Phys Act Health.

[38] Carson V, Clark M, Berry T (2014). A qualitative examination of the perceptions of parents on the Canadian sedentary behaviour guidelines for the early years. Int J Behav Nutr Phys Act.

[39] Chung H, Seo H, Forbes S, Birkett H (2020). Working from home during the COVID-19 lockdown: changing preferences and the future of work.

[40] Kim J (2018). Workplace flexibility and parent-child interactions among working parents in the US. Soc Indic Res.

[41] Patrick SW, Henkhaus LE, Zickafoose JS, Lovell K, Halvorson A, Lock S, et al. Well-being of parents and children during the COVID-19 pandemic: a national survey. Pediatr. 2020;146(4):1–11. 10.1542/peds.2020-016824 32709738

[42] Lamb ME. Father-infant and mother-infant interaction in the first year of life. Child Dev. 1977;1:167–81.

[43] Barriball KL, While A (1994). Collecting data using a semi-structured interview: a discussion paper. J Adv Nurs.

