# Supplementary material for: Perspectives of infant active play: a qualitative comparison of working versus stay-at-home parents
Source: BMC Public Health. 2021 Jan 30;21:250. doi: 10.1186/s12889-021-10286-x (PMC7847006; doi:10.1186/s12889-021-10286-x)
Supplement: Supplementary file 1 — Additional file 1. Interview Guide. The interview guide utilized in this study has been included in its entirety as a supplementary file to this manuscript. [file 12889_2021_10286_MOESM1_ESM.docx]

**PA Questions**

***Attitude Questions***

- Tell me about the feeling or thoughts you associate with when you hear the term “physical activity.”
- Tell me about the feeling or thoughts you associate with when you hear the term “active play.”
- Tell me about how you engage in active play with your infant.
- At what age is active play important for a child? Why at this age?
- What impact (if any) do you think active play has on your child at this age?
  - During the first few years of life?

***Perceived Behavioral Control***

- What recommendations for active play and sedentary time for infants have you heard previously?
  - Who did you hear this from?
  - Do you follow these guidelines? Why or why not?
- The American Academy of Pediatrics recommends parents dedicate time every day for active play and limit time spent in items that restrict movement (car seats, strollers bouncy seats.
  - What are your thoughts about these recommendations?
  - Do you think you could achieve these guidelines? Why or why not?
- Another example of recommendations are from Australia’s department of health. They recommend infants:
  - Be physically active several times in a variety of ways, particularly through interactive floor-based play-more is better. For those not yet mobile, this includes at least 30 minutes of tummy time spread throughout the day while awake.
  - Not being restrained for more than 1 hour at a time (e.g. in a stroller or high chair). Screen time is not recommended. When sedentary, engaging in pursuits such as reading and storytelling with a caregiver is encouraged.
    - Tell me your thoughts about these recommendations.
    - Do you think you could achieve these guidelines? Why or why not?
- Who has the greatest influence over your child’s activity behaviors? Why?
- If you wanted to increase the amount of active play opportunities your child receives what barriers might you face?
- What are your current activity levels?
  - How often – days/week, amount of time/day
  - Type of activities
  - How long have you been doing these activities?
  - What about your significant other?
- What are the current recommendations for adult physical activity?
- What barriers do you have to participating in physical activity?
- Who has the greatest influence over your own physical activity?
- How do you think your activity levels will influence your child?

***Subjective Norms***

- What types of physical activities does your family take part in?
  - Self, significant other, siblings?
  - What activities do you do as a family (where infant involved)?
- Has your healthcare provider talked to you about active play with your infant?
  - Have they mentioned tummy time?
- Do you know if or how your childcare provider (if applicable) utilizes active play with your infant?
  - How do you know this? (Discussion, App, Daily report, etc.)
  - Do you think your childcare provider does a good job of providing and promoting active play? Why or why not?
- Who would be the person you would most listen to when it comes to your child’s active play?
  - Why this person?
  - What about for motor development?
- Who would be the person you would most listen to when it comes to your own physical activity?
  - Why this person?
